# Some Crises in Adolescence
*A paper read to the Bristol Medico-Chirurgical Society.


**Published:** 1972-04

**Authors:** W. Lumsden Walker

**Affiliations:** Consultant Child Psychiatrist, United Bristol Hospitals


### Some Crises in Adolescence*

**W. Lumsden Walker, M.D., F.R.C.Psych.**
Consultant Child Psychiatrist, United Bristol Hospitals

### THE DECADE OF CHANGE

Adolescence may be defined as the period of life from the end of childhood, and indicated physically by the bodily changes associated with puberty, continuing until full physical development is achieved, and adult status in society is recognised. Unfortunately there is incomplete agreement as to the time-period involved. Some authors (Sandström) consider the years from 12-16, the age 10-12 being generally regarded as pre-pubertal. Others will extend the period until 18 years or later (Hadfield). It is perhaps a simpler definition, to be used in this article, that we are dealing with events normal or abnormal occurring during the second decade of life.

There is no need to consider in detail the well recognised changes occurring in the pubertal and post-pubertal period. The menarche, the changes in sexual organs, growth spurt and change in body-build including muscular development have been well described (Gessell). The change in endocrine status however which is assumed to initiate these bodily changes, is unlikely to be without its emotional concomitants, and the commonly observed adolescent lability of mood may well be related at least in part to biochemical state. We note such changes of mood at other times of endocrine change as in pregnancy or at menopause. In consequence of the overall change in physical characteristics of the body, there can be postulated a change in body-image and in control of the newly developing musculature making the adolescent and especially the boy, more clumsy just at a time of heightened self-consciousness. Most parents are well aware of sudden increase of size of their adolescent offspring, so that the sitting-room never seems large enough. Luckily this overcrowding may be off-set by the increased tendency of the adolescent to withdraw from the family group, either into solitary activities or into social activities with peers. Indeed it is a characteristic of this phase to be apparently and sometimes actually less trustful of adults and increasingly dependent upon the company and the opinions of one's contemporaries.

This leads us on to consider some of the normal emotional changes of this period of life. Throughout the first decade, if all has gone well in family life, the parents appear as the loving adults, strong, secure and apparently omniscient. In adolescence there is a 'new-look' at the parents. The adolescent is as strong as the parent and often quickly, stronger. Increasing complexity of school work, academic or technical, highlights areas of parental ignorance. Indeed there may often be a period of disillusionment in which the parent, and especially the parent of the same sex, seems to be remarkably weak and obtuse, so that the boy or girl decides that their friends have done better and they have had a most unfortunate choice in the draw. In the normal family, mutual tolerance overrides these problems, but one may see a situation now open to misunderstanding and resentment. The adolescent striving for independence is also increasingly a longed-for event which may at the same time be feared. Simultaneously the young person is becoming more aware of the implications of life and death and increasingly coming to consider his own unique individuality. The problems which may arise in consequence have been discussed in detail within the concept of a possible 'Crisis of Identity' (Erickson). This may be defined as a "conscious sense of one's individual uniqueness" — as the Self existing in one historical point in time. One thinks of the meaning of life, and the importance of one's role in life. All goes well when, in Erickson's phrase "what one feels one is and what one means to others largely coincides". Implicit in this introspection, particularly for young people studying to higher education, is a critical attitude, often positively critical, towards basic beliefs about religion, standards of behaviour in society, aims in life or other ethical problems. The flexible and tolerant parents enjoying a good past relationship with their child, can sympathetically appreciate and enjoy this phase. The parent whose rapport is already weak, and whose own life-style is founded precariously on cherished beliefs not open to criticism is likely to find that problems rapidly arise.

In such an area of conflict, the counselling of a supportive adult, if possible trusted by both generations, and himself able flexibly to appreciate the viewpoint of others and intelligently to defend essential principles will be an essential therapy. This role which may be played by priest or teacher, by family doctor or specialist demands much skill and patience.

Sexual problems are those most commonly thought of as occurring throughout adolescence. Indeed for some, very serious problems of sexual adjustment do arise. Difficulties tend to be based on misinformation concerning normal sexual behaviour or problems in relating to those of the opposite sex often, as will be seen later, based on deeper problems of relationships from earlier years. Even at the present day, anxiety may be aroused concerning masturbation or fears engendered by misinformation given by adults or contemporaries. Anxiety concerning virility and potency in the boy or sexual attractiveness and responsiveness in the girl may underlie neurotic reactions and should be excluded. However, in many cases such problems can be discussed only after good rapport has been established which may take several interviews. In simpler terms, a situation of mutual trust has to be achieved.

Other problems may arise in this decade from the

**A paper read to the Bristol Medico-Chirurgical Society.*

important pressures arising in relation to secondary education. Possible sources of stress arise from the coincidental events of puberty and move to secondary school. The child loses the one-class one-teacher, small school situation and enters a school world with subject teachers, different classrooms and an apparently vast building. For the stable child this is accepted, but for the timid; for those with difficulties in relating to peers or for those who like to please all the people all the time, the new school world presents apparently insurmountable difficulties. For the vulnerable children other problems may arise. In striving families, failure to achieve the right school, or to enter the course for the appropriate examination may induce in the child feelings of failure, intensifying problems arising later in an "identity crisis". For a few, parental and social emphasis on academic success or work success (the 'rat-race') may induce the feeling of "being valued for what you can do, not for what you are". This can be destructive of self-esteem, or in those constitutionally more self-assertive may lead to revolt against expected standards and a seeking of alternative satisfactions (the teen-age rebel, or at worst "drop out"). Indeed when parent/child relationships are already weak or bad, there may be complete rejection of parental ideals, or hostility expressed in anti-social conduct disorders. Finally, for the less bright or the socially deprived, who find school of limited value, the work may seem irrelevant and boring and problems of control and discipline may arise (Clegg). I have attempted to summarise some of the more important changes to which the child is subjected on entering this period of life. We may note that discussion of possible crisis situations has tended to stress pre-existing personality state and family relationships. We must therefore look more closely at these primary determinants of behaviour.

### PRE-MORBID PERSONALITY

This term is used in psychiatric assessment when formulating both diagnosis and prognosis. Entering the potential stress of adolescent change, it will be the pre-existing personality strengths or weaknesses which mainly determine whether the adolescent is one of the approximately 85% who pass through this phase without major difficulty or one of the 15% who suffer pain themselves or cause pain to others.

### CONSTITUTION

Whatever we may mean by this ill-defined term, it must play some part. I take it to indicate the probability that there is a degree of genetically determined vulnerability to breakdown under stress. Amongst other authors Rutter has shown that psychiatric illness in childhood, excluding psychotic illness, tends to present symptomatically either as sickness painful to the child as in neurotic symptoms especially of anxiety, depression or phobic or obsessional states. Alternatively the illness presents as causing pain to the world outside—as in anti-social acts of hostility, abuse, vandalism, rebellion or delinquency. In some there is an alternation between the two modes, particularly in suicidal threat or act coupled to depression alternating with serious conduct disorder, the hostility generally directed at the parents. It is postulated that there is a constitutional predisposition to produce in time of stress, either mainly neurotic or mainly "acting-out" symptoms. However these constitutional factors in my opinion are of greater weight in determining the form of breakdown, rather than being causative in themselves.

Developmental aspects of personality although fully discussed elsewhere (Hadfield) (Miller), are crucial and must be briefly discussed.

1. **Self-esteem,** which may be loosely equated with the Freudian concept of ego-strength, is an essential basis to a future acceptance of ones own individual worth. It is probably a consequence of unconditional acceptance of the child by his parents as worthy of love in his or her own right, this feeling demonstrated in an infinite number of minor events or attitudes, spoken or even more importantly, unspoken. The most critical period is the first three to four years of life. Subsequently repair of damaged self-esteem is slow and difficult.

2. **Incorporation of Parental Characteristics and Standards** again is a gradual process occurring slowly over the first five or more years of life. The parent of either sex forms the model not only for functioning as culture expects as male or female, but also as model for the future choice of partner (Sears). Parental cohesiveness encourages positive feelings and attitudes of security. Parental discord, parental absence or hostility militate against adequate personality development, or acceptance of normal social standards. Occasionally of course, a child may successfully identify with the standards of a family which itself rejects the normal standards of society. Since the family may be seen in sociological terms as the 'Cradle of the Culture' responsible for transmitting the best features (one hopes) to the next generation, then the enormous gains from being reared with affection by mutually supportive parents becomes clear. Vulnerable children, that is those 'at-risk' of breakdown, will be found in large numbers in unhappy families or families unable to function through external stresses (Gould). It follows also that when substitute parental care becomes necessary this must be given by the same people, over a long period of time. The child receiving even the best of institutional care, where staff changes occur often only too frequently, also enters adolescence as vulnerable. Further examples will occur to the commonsense of the reader.

3. **Role functioning in the family** is less commonly considered as of consequence and I must explain the term. There is a tendency to 'labelling' within families, so that individual children are expected to function in a certain way, and indeed are very likely to play the part assigned to them. As for example children implicity, or sometimes explicitly referred to as the 'helpful' child, the 'clever one', the 'sympathetic', the one who 'always helps mum', or the more negative 'unhelpful', 'naughty', 'delicate', 'sick', and so forth. The experienced family doctor will have seen this game played by adults themselves as well as induced in the children. Less readily noted is the 'scapegoat'. At its most abnormal, this term implies that the bad or hostile feelings of the family are projected on to and then expressed by one member. Often this is demonstrated when one child is removed from the family circle (perhaps to a boarding school or to a relative or perhaps to reformatory) and almost immediately a new member becomes 'bad'. The rest of the family can only function so long as somebody else is to blame. (Likewise there can be neighbourhoods in which one family plays this role for the community—"however low you sink you are never as bad as the Walkers!") More seriously the child seems to suffer most when adult feelings of guilt are projected on to the child, who accepts the role and becomes guilty and periodically ill (sick) or delinquent (bad) the response often determined by innate constitutional factors.

4. **The Cultural Characteristics of the neighbourhood,** usually accepted, occasionally rejected, are a final factor in determining the patterns of response in adolescence. At a very simple level it will be obvious that in some neighbourhoods, example will lead to the probability of psychosomatic illness or neurotic anxiety becoming the more acceptable abnormality while in others, patterns of delinquency may be the outward expression of inner tensions.

### SOME COMMONER CRISIS EVENTS

Our adolescent who is passing through this period of change, will usually take the phase in his stride, but some may have problems. In the great majority of cases these will yield to support and counselling from home, or from friendly adults, professional or other. A small but important minority (10-15% probably) will have greater problems and will produce major sickness or disturbance necessitating skilled intervention. Of such problems I propose to discuss some of those more likely to arise. The possibility that the adolescent will respond to wise support from an adult whom he can trust and who is not a member of the family offers the doctor a unique opportunity for therapy. However we must remember that the adolescent is still a member of his family (and indeed until at least 16, still a schoolchild). Work with a 'teen-ager' must almost always be combined with work with the family, and indeed many therapists successfully use combined family groups in therapy. This however needs experience and has its own particular problems. Extended education to 18 or older, and the use in major environmental problems of the 'Care Order' to 18 prolongs in our present day, the period of dependence, abruptly now terminated legally at this age.

### SOME PSYCHIATRIC PROBLEMS

1. **School Refusal.** The maximum incidence is at secondary school age. Distinction must be drawn between **Truancy** and neurotically determined **School Phobia.** Briefly the truant is likely to be off school without the knowledge of the parent, is probably retarded in school work, and is away from home wandering the streets often in the company of fellow truants. The situation is a common precursor of delinquency. The child is not infrequently emotionally deprived. Occasionally the child is absent from school with the consent of the family who see school as irrelevant. This last sub-group is not 'sick' in any sense.

**School Phobics** refuse to go to school despite all efforts of the family. They remain at home, can offer no explanation for the refusal and if once got to school appear quite happy. Often there is an anxious and over-involved mother and a sick or inadequate or absent father. If attempts are made to force the child to school he or she will resist with force (e.g. helpful neighbour or doctor kicked and bitten by normally "nice" child). Parent and child should be separated, getting the child to a 'protected' environment temporarily if necessary (e.g. special class, Neurosis unit as day patient etc.). Tranquillisers play a small part, and if used must be given on waking about 7 a.m. Chlorpromazine 25-50 mg or Diazepam 5-15 mg are most usual. Therapy is aimed at the management of the child and parents simultaneously and there is need for the help of a skilled psychiatric social worker. Delay in starting treatment reinforces the anxiety of both child and parents and early specialist help is advisable.

2. **Depressive illness** is common in childhood and adolescence. Diagnosis may be difficult as mood may change rapidly in the adolescent and the typical adult picture need not be seen. Disturbance of sleep and concentration may occur. Loss of appetite and loss of weight are less common. Depression may present as school phobia or as psychosomatic illness, often headache or abdominal pain. The youngster has feelings of inadequacy or failure, or feels unloved or under-valued. Family or school crisis, or a sudden break in a friendship, particularly a boy/girl affair may precipitate a severe depressive mood swing, sexual problems of adjustment may be present. **Impulsive suicidal gestures** are less rare than is usually thought. One must always enquire for suicidal thoughts. One author (Fromm) places greater emphasis on anti-depressants than most psychiatrists accept. I rarely use MAO drugs as control of diet is unreliable at this age and for the more anxious we may add to the family worry. Tricyclic anti-depressants (75-100 mg daily, divided in two doses) are worthy of trial but if they are going to act will do so in two to three weeks. All medication must be dispensed in small quantities as an impulsive overdose is not uncommon in a child in a mood of despair. Indeed despair, rather than depression of adult type, is the more common adolescent picture. Emphasis in treatment is investigation, and attempted correction of underlying family, school or psycho-social problems, together with continuing supportive psychotherapy, to restore self-esteem. If the child is on medication while attending school, the teacher should be advised lest the patient be in trouble because he is lethargic or drowsy. Alternatively when the depressive response may be irritability or resentment, advice to the school and school doctor or nurse can be helpful to avoid increasing the child's problems.

3. **Obsessional Illness.** This will be seen rarely in an individual practice, but is not uncommonly seen in outpatients. The picture is found in older adolescents often under stress of higher examination or settling in a new job. The pattern is typical—as seen in adult illness. The behaviour is compulsive and causes distress to the patient and the family. The pre-morbid personality is commonly overconscientious and obsessional and there may be a family history. Not uncommonly a parent colludes in the symptoms (e.g. compulsive washing to get rid of "dirt", compulsive reading or repetition of phrases, or rituals for dressing or undressing). Careful and tactful investigation will sometimes reveal underlying sexual anxieties and reassurance will help. Psychotherapy will help through the crisis. The illness is often terminated for a time, when the worrying event (e.g. examination) is over. Of medication, phenothiazines are most helpful, but if the patient is made too drowsy to work, they will increase anxiety and so are refused or not taken. The short-term prognosis is good, the long-term more guarded. In the short-term the case will present as a crisis since parents complain that the child 'never gets to bed' or hour-long washing or dressing rituals cause lateness in getting to school and precipitate school refusal or attitudes of despair. Immediate supportive psychotherapy is the real answer to mitigate the crisis, although rarely to cure. If possible, the whole family should be involved.

4. **Runaway reaction:** This is less clearly a neurotic response in the usual sense, but is a crisis event in adolescence. Running away from home or boarding-school is 'newsworthy' and pressure from newspapers may add to the family problems. It is akin to suicide often, inasmuch as it is a 'cry for help' or flight from what is felt to be an intolerable situation. The youngster is, of course, while on the run, at risk of becoming involved in delinquency, or exploited sexually. I regard it in the majority of cases as an indication for urgent psychiatric investigation. A useful discussion is found in a recent paper (Jenkins).

5. **Delinquency.** This presents as a 'medical' emergency usually when the middle-class parent seeks advice concerning rebellious behaviour in their adolescent son or daughter. It may be part of an identity-crisis, or may be an aggressive response to controls from a parent seen as unaffectionate and critical, or disinterested. Misuse of drugs or alcohol, school failure, theft particularly of motor vehicles and hostility to school authority are common presenting events in the boy. When the child is not living in a high-delinquency neighbourhood, and when parents are not criminal, then careful investigation of the family picture is called for. Moral judgements have no place. An attempt to gain the confidence of the youngster is essential. This is even more the case with the girl more likely to present as 'beyond control' — staying out late, frequenting undesirable clubs or cafés, and sexually promiscuous (or claiming to be). In either sex such conduct may be an act of aggression aimed at the parents (the attitude of neighbours causes the parent to suffer), or may be a form of "social suicide". Often the patient is of very low self-confidence and self-esteem and has labelled himself in our jargon as a 'bad object'. Not infrequently depression or despair may alternate with anti-social conduct. Often the parents are very preoccupied with their own careers, own problems or own illnesses and this egocentricity may make it essential to focus attention and help on the child. Psychotherapy can be very rewarding.

I must conclude this outline by stressing that I have discussed only some adolescent crises. Time and space limit further aspects, but the references quoted will suggest further reading. In all cases patience is the rule, and careful history-taking even when one thinks one knows the family. The help of the social worker is often essential since in general the adolescent in difficulties is mistrustful of adults and suspects that what he says will be related back to the parents with whom the doctor is seen as colluding. Commonly therefore separate workers (but working in close association) must work with child and with parents. Under the age of fifteen to sixteen however the emphasis in **all** cases is on family therapy and I may conclude by quoting from T. S. Eliot

> "Indeed it is often the case that my patients
> Are only pieces of a total situation
> Which I have to explore. The single patient
> Who is ill by himself is rather the exception."

### REFERENCES

1. SANDSTROM, I. (1966). The Psychology of Childhood and Adolescence. Pelican Books.
2. HADFIELD, J. A. (1962). Childhood and Adolescence. Pelican Books.
3. GESSELL, A.. ILG, F. L. and AMES, L. B. (1956). Youth: the years from Ten to Sixteen. Hamish Hamilton.
4. ERICKSON, Eric (1968). Identity, Youth and Crisis. Faber and Faber.
5. CLEGG, A. and MEGSON, B. (1968). Children in Distress. Penguin Books.
6. RUTTER, M. (1965). Classification and Categorization in Child Psychiatry. Journal of Child Psychology and Psychiatry **6,** 2. 71.
7. MILLER, D. (1969). The age between: Adolescents in a disturbed society. Cornmarket/Hutchinson.
8. FROMM, E. A. (1968). Depressive illness in Childhood, in: Recent Developments in Affective Disorders (Ed. Coppen & Walk). British Journal of Psychiatry. Special Publication No. 2. Headley Brothers.
9. JENKINS, R. L. (1971). The Runaway Reaction. American Journal of Psychiatry **128**, 168-173.
10. ELIOT, T. S. The Cocktail Party.

